# Current Understanding of Hypoxia in Glioblastoma Multiforme and Its Response to Immunotherapy

**DOI:** 10.3390/cancers14051176

**Published:** 2022-02-24

**Authors:** Jang Hyun Park, Heung Kyu Lee

**Affiliations:** Graduate School of Medical Science and Engineering, Korea Advanced Institute of Science and Technology (KAIST), Daejeon 34141, Korea; janghyun.park@kaist.ac.kr

**Keywords:** glioblastoma multiforme (GBM), hypoxia, antitumor immunity, immunotherapy

## Abstract

**Simple Summary:**

Glioblastoma multiforme (GBM) is the most aggressive tumor type in the central nervous system. Hypoxia, defined as a lack of sufficient oxygen in tissues, is the most detrimental factor for the survival of GBM patients, promoting drug resistance, and invasion and inhibition of immune responses. Traditionally, tumor hypoxia has been studied from a narrow viewpoint, excluding the immune system and focusing primarily on the effect of hypoxia on blood vessels and tumor cells. More recently, however, evidence highlighting the important role of immunosurveillance has been uncovered for multiple tumors, including GBM. Thus, connecting the knowledge gained from traditional hypoxia studies with findings from recent immunological studies is urgently needed to better understand the role of hypoxia in cancer.

**Abstract:**

Hypoxia is a hallmark of glioblastoma multiforme (GBM), the most aggressive cancer of the central nervous system, and is associated with multiple aspects of tumor pathogenesis. For example, hypoxia induces resistance to conventional cancer therapies and inhibits antitumor immune responses. Thus, targeting hypoxia is an attractive strategy for GBM therapy. However, traditional studies on hypoxia have largely excluded the immune system. Recently, the critical role of the immune system in the defense against multiple tumors has become apparent, leading to the development of effective immunotherapies targeting numerous cancer types. Critically, however, GBM is classified as a “cold tumor” due to poor immune responses. Thus, to improve GBM responsiveness against immunotherapies, an improved understanding of both immune function in GBM and the role of hypoxia in mediating immune responses within the GBM microenvironment is needed. In this review, we discuss the role of hypoxia in GBM from a clinical, pathological, and immunological perspective.

## 1. Introduction

Tumor cells have distinct metabolic features compared to normal cells. For example, although normal cells usually suppress glycolysis under normoxic conditions (i.e., the Pasteur effect), tumor cells preferentially use glycolysis despite the presence of oxygen, a phenomenon known as the Warburg effect [1]. Lactic acid accumulation resulting from the Warburg effect is metabolic hallmark of the tumor microenvironment (TME), leading to low pH. Critically, these unique metabolic characteristics can inhibit antitumor immune responses, making the TME more favorable for tumor progression [2]. Thus, a precise understanding of metabolic programming within the TME is essential for the development of effective antitumor therapy.

Oxygen is the most basic and important component of cellular metabolism. Many enzymes, such as oxygenase, require oxygen for their function [3], and large amounts of energy are generated by oxidative phosphorylation compared to glycolysis [4]. A lack of oxygen leads to hypoxia—a hallmark of many cancers that is linked to tumor progression and worse clinical outcomes for patients [5]. Hypoxia in cancer can result from the fast proliferation of tumor cells; this causes some tumor cells to be located far from oxygen-supplying blood vessels (>180 μm) [6], leading to limited oxygen diffusion. In addition, the TME often promotes angiogenesis, which can result in the formation of abnormal, closed blood vessels, further inducing hypoxia [7]. Critically, the presence of hypoxia reprograms tumor cells through multiple proteins, such as hypoxia-inducible factor (HIF)-1α. Such hypoxia-adapted tumor cells are more invasive and resistant to therapies, and they can also evade immunosurveillance.

Beyond tumor cells themselves, tumor-infiltrating immune cells are also under hypoxic conditions in the TME. Normoxic ambient air contains 21% O_2_, whereas O_2_ concentrations of 2–9% (14.4–64.8 mmHg) are present in tissue under physiological normoxia. In contrast, some parts of normal organs, such as the bone marrow and thymus, as well as the TME, are hypoxic, containing approximately 1% O_2_ (7.2 mmHg) [8]. A number of studies have investigated immune cell function in the context of hypoxia; however, these have reported contradictory results regarding whether hypoxia promotes beneficial or adverse effects on immune cells. For example, CD8^+^ T cells activated under hypoxia show stronger cytotoxic effects against tumor cells than those activated under normoxia [9]. Similarly, natural killer (NK) cells cultured under hypoxic conditions following normoxia are more highly activated than normoxic NK cells [10]. Conversely, hypoxia downregulates interferon (IFN)-γ production by CD8^+^ T cells under T cell receptor (TCR) stimulation and attenuates NK cell-mediated cytotoxicity [11,12]. Intriguingly, NK cell activity is enhanced by short-term, but not long-term, hypoxia [13,14], and continuous activation of CD8^+^ T cells under hypoxia promotes characteristics distinct from those present in acutely activated CD8^+^ T cells [15]. This suggests that while a HIF-1α-mediated glycolytic burst enhances the activity of cytotoxic cells, mitochondrial dysfunction in response to long-term hypoxia attenuates cytotoxic and inflammatory functions. Overall, these complex hypoxia-associated phenotypes are dependent on numerous different factors and experimental conditions, suggesting that understanding the role of hypoxia in antitumor immunity is likely to be more complicated than expected.

Of all the organs in the body, the brain requires the highest amount of oxygen. Thus, although the brain comprises only 2% of total body weight, it consumes 20% of the body’s oxygen. Oxygen levels in the brain differ depending on the region. For example, the oxygen level in the midbrain is approximately 0.5%, whereas in the pia, it is 8% [16]. Brain tumors have been shown to contain 1.25% O_2_, with the peritumoral area containing 2.5% O_2_ [17]. Thus, brain tumor regions are mostly hypoxic compared to normal brain tissue. Glioblastoma multiforme (GBM), the most aggressive brain tumor type, can be classified as a hypoxic tumor. Irradiation is the most frequently used antitumor therapy for brain tumors; however, hypoxia-mediated stemness promotes cellular resistance to irradiation. Thus, studies aimed at understanding the effects of hypoxia within brain tumors are urgently needed. In this review, we discuss recent findings on the role of hypoxia in tumor biology and in antitumor immunity against brain tumors.

## 2. Glioblastoma Multiforme (GBM)

### 2.1. GBM

Tumors of the central nervous system (CNS) are relatively rare compared to other peripheral tumors, with CNS tumors showing an estimated frequency of about 1% amongst those detected in all tumor sites [18]. Most CNS tumors, about 70% of cases, are non-malignant, half of which are meningiomas. In 30% of cases, however, CNS tumors are malignant, with glioblastomas accounting for about 50% of all CNS malignancies [19]. Despite its low overall incidence, glioblastoma is an important tumor type due to the high average years of life lost from this cancer, which amounts to 20.1 years [20]. In addition, according to a report from the Australian Institute of Health and Welfare (AIHW), between 1988–1992 and 2013–2017, the estimated percentage change in the 5-year survival rate for brain tumor was only about 2.3%, whereas all cancers combined showed an increase of 18.3% [21].

Most GBMs are primary tumors, although a small portion develop from low-grade astrocytoma and thus are known as secondary GBM [22]. A study by Verhaak et al. [23] further reported that GBM can be divided into four subtypes based on gene expression patterns: classical, proneural, neural, and mesenchymal. The classical type includes amplifications of chromosome 7 and epidermal growth factor receptor (EGFR), as well as a homozygous deletion spanning the *Ink4a/ARF* locus. The mesenchymal type shows high expression of chitinase-3-like protein 1 (CHI3L1) and tyrosine-protein kinase Met (c-Met), with neurofibromatosis type 1 (*NF1*) mutation/deletion or low expression of NF1. Mesenchymal type GBM also displays a higher percentage of necrosis and inflammation than other types. The proneural type has platelet-derived growth factor receptor A (PDGFRA) abnormalities and mutations on both tumor protein p53 (*TP53*) and isocitrate dehydrogenase 1 (*IDH1*), similar to secondary GBM. In contrast, the neural type is similar to normal brain tissue in terms of the gene expression pattern. Recently, however, the Verhaak group suggested that the neural GBM subtype might, in fact, be contamination from normal brain tissue [24]. Because *IDH* mutation is associated with better prognosis, proneural subtype GBM is thought to be associated with better patients outcomes, whereas mesenchymal subtype GBM is associated with worse outcomes [25].

### 2.2. Clinical Approaches for GBM

To improve the survival of GBM patients, maximal resection of tumor tissues is recommended. This reduces mass effect and enhances efficacy of adjuvant therapies, leading to increased survival rates [26]. However, despite the rapid development of improved detection methods, complete resection is often difficult due to the presence of complex vasculature, location of the tumor, and fear of damage to intact brain tissues. Thus, in many cases, resection is ineffective, and recurrence after surgery is common [27].

**Figure 1:**
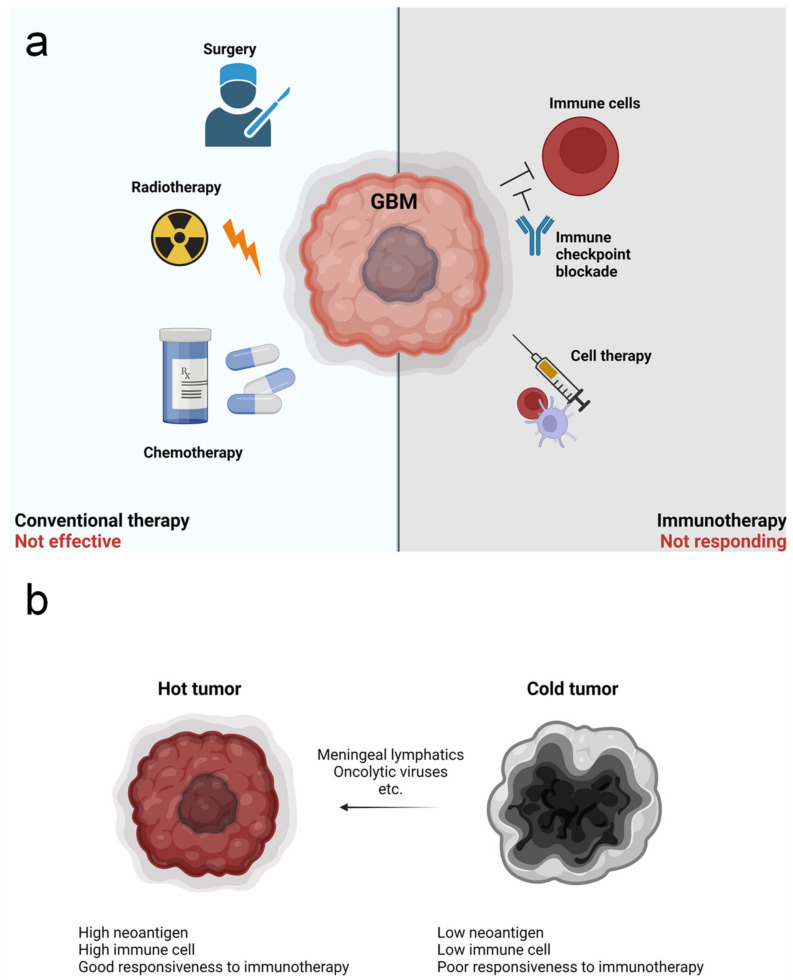
Therapeutic approaches for glioblastoma multiforme (GBM) and hurdles to treatment. (**a**) There are multiple strategies to care for GBM patients. Conventional GBM therapy involves surgery, followed by radiotherapy and concomitant chemotherapy. However, this is not fully effective. Recently, immunotherapies have been developed and shown promising results for other tumors. Immune checkpoint blockade approaches for inhibiting immunosuppression, and cell therapies, such as dendritic cell (DC) vaccines, are now being tested for GBM. However, responsiveness to these therapies is poor. (**b**) Tumors can be classified as “hot” or “cold”. Hot tumor shows high levels of neoantigens, increased infiltration of immune cells, and better responsiveness to therapies relative to cold tumors. Thus, several approaches for converting cold tumors into hot tumors, such as by manipulating lymphatics or through the use of oncolytic viruses, are being studied.

Current standard care for GBM is Stupp’s regimen, which involves radiotherapy (2 Gy per day, 5 days per week, up to a total of 60 Gy), with concomitant temozolomide (TMZ) treatment (Figure 1a) [28]. Similar to other chemotherapies, TMZ induces DNA damage via methylation of O6 and N7 positions on guanine and the N3 position on adenine, which promotes cell death. Sensitivity to TMZ treatment is largely dependent on methylation of the O6-methylguanine-DNA methyltransferase (*MGMT*) promoter [29]. MGMT repairs DNA, and consequently, patients with *MGMT* methylation, which inhibits expression of this gene, are sensitive to TMZ treatment [30]. In 2015, a device delivering alternating electric fields, also known as tumor-treating fields (TTFs), was approved by the US Food and Drug Administration for GBM patients [31]. In phase 3 trials, this device promoted a significant increase in overall survival (OS) and progression-free survival (PFS) compared to TMZ alone [32].

Complex vasculature is also highly associated with GBM progression, and therefore, anti-angiogenesis therapies, including bevacizumab, have been tested. In phase 3 trials, bevacizumab treatment improved PFS; however, disappointingly, a significant improvement in OS was not observed [33,34]. Furthermore, long-term treatment with bevacizumab is associated with increased hypoxia and invasiveness [35]. Alternatively, vessel normalization via TIE2 activation and angiopoietin-2 (ANG2) inhibition showed promising results in rodent models, leading to less hypoxia and invasion, although this strategy has not been tested in humans [36].

In recent years, the emergence of cancer immunotherapy was expected to be a game changer for treatment of various tumor types, including GBM. Toward this goal, many strategies have been suggested, such as dendritic cell (DC) vaccines, immune checkpoint inhibitors, chimeric antigen receptor (CAR) T cell therapy, and adoptive cell therapy. Although none of these have shown promising results for GBM patients, a massive number of studies remain ongoing [37]. Currently, the most popular strategy for GBM immunotherapy is immune checkpoint blockade, such as with anti-programmed cell death 1 (PD-1) therapy. However, recent phase 3 clinical trials using nivolumab in unmethylated-MGMT GBM with radiotherapy and methylated-MGMT GBM with chemoradiotherapy did not show any improvement in OS and PFS [38]. Although it is too early to judge, many concerns regarding the use of immunotherapy for GBM exist due to the fact that GBM is classified as a “cold tumor” with poor immune cell infiltration [39]. Thus, various strategies for converting GBM into “hot” tumor have been suggested, including the use of oncolytic viruses and/or manipulating meningeal lymphatics (Figure 1b) [40,41].

## 3. GBM and Hypoxia

### 3.1. Histological Characteristics of GBM

**Figure 2:**
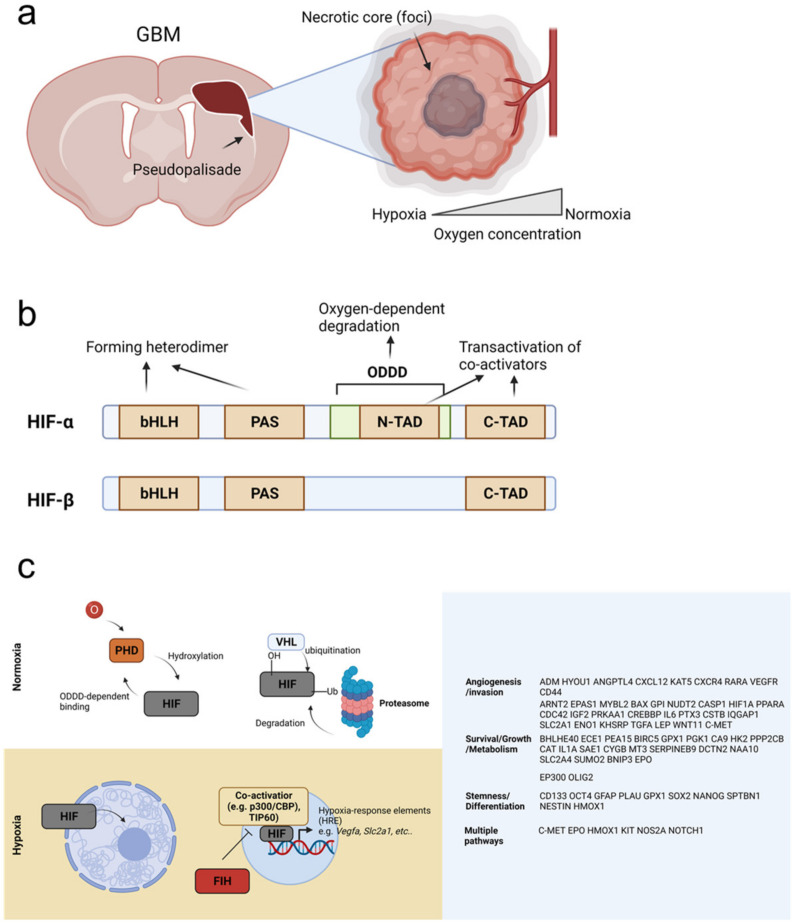
Responses to hypoxia in GBM tumors. (**a**) GBM tissue shows aggressive invasiveness and pseudopalisades. Oxygen is supplied by blood vessels, and thus, tumor cells located far from vessels become hypoxic due to poor oxygen diffusion, forming necrotic core (foci). Tumor cells that escape from hypoxia form pseudopalisades. (**b**) Hypoxia-inducible factor (HIF)-α and HIF-β have basic helix–loop–helix (bHLH), Per–Arnt–Sim (PAS), and C-terminal (C-TAD) domains. The bHLH and PAS domain are responsible for forming the heterodimer, and the C-TAD domain promotes transactivation of co-activators. HIFα also has an N-terminal (N-TAD) domain and an oxygen-dependent degradation domain (ODDD), which mediate its oxygen-dependent degradation via the ubiquitin–proteasome pathway. The N-TAD also participates in transactivation of co-activators. (**c**) Under normoxia, oxygen-dependent prolyl hydroxylase (PHD) enzyme is active and binds to HIF in an ODDD-dependent manner. PHD hydroxylates HIF, which allows von Hippel–Lindau (VHL) to bind and recruit E3 ubiquitinase. This enzyme ubiquitinates HIF, targeting it for binding and degradation by the proteasome. In contrast, under hypoxia, HIF is stable and translocates into the nucleus, where it binds to co-activators, such as p300/CBP or TIP60, and turns on expression of hypoxia-response element (HRE) genes. HIF can regulate multiple cellular processes via activation of these HRE genes. HIF-suppressors, including factor-inhibiting HIF (FIH), function to inhibit binding between HIF and its co-activators and block HRE activation.

GBM tumors display unique features, such as necrotic foci, pseudopalisades, and microvascular hyperplasia (Figure 2a), which are thought to be important for the fast growth and invasiveness of GBM cells [42]. Pseudopalisades may result from the migration of tumor cells escaping from a hypoxic region to form the invasive front edge. Pseudopalisading cells shape microvascular hyperplasia, forming tuft microaggregates around the edge of blood vessels and leading to the formation of glomeruloid bodies [43]. These features are largely mediated by angiogenesis-induced hypoxia. For example, excessive expression of vascular endothelial growth factor (VEGF) induces the hyper-proliferation of endothelial cells, resulting in defective and permeable blood vessels that can be easily disrupted [44]. This abnormal vasculature in the GBM microenvironment inhibits the delivery of oxygen, as well as drugs and immune cells [36]. In addition, hypoxia resulting from abnormal vessels promotes the invasion of tumor cells, a main hurdle for therapies against GBM [42].

### 3.2. Cellular Sensing of Hypoxia

Cells can sense the surrounding oxygen level through multiple molecular mechanisms. Of these, the most well-studied is the highly conserved HIF pathway [42], which acts as the major oxygen-sensing pathway in metazoan species [45]. The transcription factor HIF is a heterodimer formed from two distinct subunits, HIFα and HIFβ. In humans, HIFα has three isoforms. HIF-1α is ubiquitously expressed and overexpressed by tumor cells [46,47]. In contrast, HIF-2α is expressed in distinct cell populations, such as in subsets of tumor-associated macrophages (TAMs) [48]. HIF-3α is also selectively expressed, although its expression in immune cells is not clear [49,50]. Target genes for HIF-1α and HIF-2α show some degree of overlap; however, a subset of genes is distinctly regulated by each transcription factor [51]. HIF-3 can also function as a transcriptional activator for a unique set of genes [52], although it is most commonly known to be a dominant-negative regulator of HIF-1, due to its lack of a C-terminal (C-TAD) domain [53]. In contrast, the aryl hydrocarbon receptor nuclear translocator (ARNT), HIF-1β is expressed ubiquitously [42].

HIF-1α, HIF-2α, and HIF-1β all contain a basic helix–loop–helix (bHLH) domain, a Per–Arnt–Sim (PAS) domain, and a C-TAD domain. HIF-α also has additional oxygen-dependent degradation domain (ODDD) and an N-terminal (N-TAD) domain. The bHLH and PAS domains form the heterodimer and bind to hypoxia-response elements (HREs) [54], whereas the C-TAD and N-TAD domains are involved in transactivation of coactivators, such as p300/CBP (Figure 2b) [55].

In normoxia, HIFα is bound to prolyl hydroxylase 1–3 (PHD1–3) via the ODDD, and PHD hydroxylates two prolyl residues of HIFα. PHD is regulated by O_2_ levels due to its 2-oxoglutarate-dependent and iron-dependent dioxygenase domains [56]. Hydroxylation of HIFα allows it to bind von Hippel–Lindau (VHL), which recruits E3 ubiquitin ligases. These promote the ubiquitination of HIFα and its subsequent degradation by the proteasome [57]. Conversely, under hypoxic conditions, PHD activity is lost, and stable HIFα translocates into the nucleus, where it binds HIFβ and coactivators, such as p300/CBP (Figure 2c) [58]. Factor-inhibiting HIF (FIH), an O_2_-dependent hydroxylase, also functions in HIFα regulation by blocking HIFα binding to coactivators [59]. HIF target genes are reviewed in detail elsewhere (e.g., [60]) (Figure 2c).

### 3.3. HIF, HRE Genes, and GBM

As noted above, oxygen-dependent gene expression is mainly mediated by HIF and downstream HRE genes. The HIF-dependent hypoxic response regulates multiple cellular activities, including metabolism, migration, angiogenesis, and differentiation [61], and this pathway promotes invasiveness in hypoxic GBM cells via multiple mechanisms. For example, carbonic anhydrase 9 (CA9), a zinc-dependent enzyme that catalyzes the conversion of CO_2_ into bicarbonate, is known to be affected by hypoxia and is highly expressed in GBM cells [62]. Hypoxia also stabilizes the EGFRvIII protein by promoting interaction with integrin β3 in GBM cells [63] and further induces recruitment of the integrins αvβ3 and αvβ5 to the surface of GBM cells, leading to activation of focal adhesion kinase (FAK) [64]. Procollagen-lysine 2-oxoglutarate 5-dioxygenase 2 (PLOD2), an enzyme that regulates collagen cross-linking, is also controlled by hypoxia in a HIF-1α-dependent manner [65]. Collectively, these protein interactions promote invasion of GBM cells. In addition, epithelial-to-mesenchymal transition (EMT)- and metastasis-related genes, such as recombination signal binding protein for immunoglobulin kappa J (RBPJ) [66], zinc finger E-box-binding homeobox 1 (ZEB1) [67], and Twist-related protein 1 (TWIST1), are known to be regulated by hypoxia via the HIF-1α pathway [68]. Hypoxia has also been reported to induce expression of C-X-C chemokine receptor type 4 (CXCR4) in GBM cells and of CXCL12 in endothelial cells, and both C-C motif chemokine receptor type 5 (CCR5) and C-C motif chemokine ligand 4 (CCL4) are positively regulated by hypoxia [69,70,71].

Several key pathways are involved in cellular adaptation to hypoxia. For example, hypoxia suppresses cap-dependent protein translation at the level of translation initiation [72]. This suppression is primarily regulated by protein kinase R (PKR)-like endoplasmic reticulum (ER) kinase (PERK) and the mechanistic target of rapamycin (mTOR) complex 1 (mTORC1) [73]. However, some genes are continuously translated under hypoxia, such as those associated with stress response. These commonly include genes related to antioxidant response, amino acid transport, metabolism, and autophagy. Further, when activated PERK phosphorylates eIF2α to induce translational suppression [74], the remaining ribosomes are able to translate mRNAs encoding proteins for unfolded protein response (UPR), such as ATF4 [75]. In addition, activation of inositol-requiring transmembrane kinase/endoribonuclease 1α (IRE1α) in response to hypoxia promotes activation of functional X-box binding protein 1 (XBP1), which regulates multiple metabolic pathways [76,77]. Mitochondrial functions are also regulated by hypoxia, as 0.3% O_2_ is the rate-limiting threshold for electron transport complex (ETC) activity [78]. Likewise, the tricarboxylic acid (TCA) cycle is reduced, mitochondria translocate to the perinuclear site, and mitochondrial fission and mitophagy are induced in both a HIF-dependent and HIF-independent manner [79,80,81,82]. The most prominent aspect of adaptation to hypoxia is upregulation of glucose uptake and glycolysis. This is mediated by HIF-1α, which directly regulates expression of glucose transporter 1 (GLUT1), GLUT3, hexokinase 1 and 2, enolase 1, phosphoglycerate kinase 1 (PGK1), pyruvate kinase M2 (PKM2), lactate dehydrogenase A (LDHA), and phosphoinositide-dependent kinase 1 (PDK1) [83,84]. PDK1 inhibits conversion of pyruvate to acetyl CoA, thereby promoting lactate production [85]. This glycolysis-mediated enrichment of lactate and H^+^ ions lowers the surrounding pH, and critically, both the presence of lactate and low pH are harmful for antitumor immunity [86]. Hypoxia adaptation mechanisms are also involved in cell death pathways. For example, hypoxia induces autophagic cell death in apoptosis-competent cells via BCL2-interacting protein 3 (BNIP3) [87] and promotes necrosis of neuronal cells [88]. However, alarmin release by necrotic cells further promotes progression of glioblastoma stem-like cells [89].

Critically, hypoxia is also associated with increased radioresistance in GBM. Although the underlying mechanism is not clear, several molecular pathways have been implicated in this phenomenon. In one instance, it was shown that the mitogen-activated protein kinase (MAPKK; MEK)/Extracellular signal-regulated kinase (EKR) pathway promotes hypoxia-mediated radioresistance via the activity of DNA-dependent protein kinase, catalytic subunit (DNA-PKcs) and HIF-1α [90]. Another study found that phospholipase C gamma (PLCγ) binding to fibroblast growth factor receptor 1 (FGFR1) induces protein kinase C (PKC) activation in response to HIF-1α regulation, and this also induces radioresistance [91]. In addition, hypoxia promotes glioma stem cell (GSC) formation by inducing stem cell marker genes, including octamer-binding transcription factor 4 (OCT4), NANOG, SRY-Box transcription factor 2 (SOX2), Kruppel-like factor 4 (KLF4), and cMYC, while downregulating expression of glial fibrillary acidic protein (GFAP) [92,93], and this was further shown to be critical for inducing radioresistance.

HIF proteins and HRE genes also regulate a number of angiogenesis-related molecules, such as VEGFs, placenta growth factors (PGFs), angiopoietin (ANGPT), CXCL12, and platelet-derived growth factor B (PDGF-B) [94]. In response to hypoxia, VEGFs and PGFs bind to VEGF receptor (VEGFR)-1 and -2 on endothelial cells and induce proliferation and survival via the ERK/PI3K/AKT pathways [95]. Rho GTPase-mediated migration and membrane type matrix metalloproteinase (MMP)-mediated extracellular matrix (ECM) degradation are also induced by VEGF/PGF binding [96,97]. Among the angiogenesis-related molecules induced by hypoxia, the most well-studied protein is VEGF-A. Notably, although this protein is critical for homeostatic vasculature, hypoxia-mediated VEGF-A overexpression induces vascular permeability, which in turn, inhibits the delivery of drugs and immune cells, limits perfusion, and even further promotes hypoxia [94].

## 4. Hypoxia and Antitumor Immunity

### 4.1. Hypoxia in an Immunological Niche

Like other cells, immune cells need proper oxygen levels for survival and function. However, despite mechanisms to maintain homeostasis and normoxia in most instances, some niches can become hypoxic due to anatomic characteristics of organs or burst of cellular expansion. This is referred to as “physiological hypoxia” [50], and in some cases, it is necessary for proper organ function. The bone marrow is one of the most well-characterized physiologically hypoxic organs [98]. Here, hypoxia is critical for maintaining hematopoietic stem cell (HSC) homeostasis [99]. Although it remains controversial, HIF-1β was shown to be required for quiescence, survival, and development of HSCs, whereas HIF-2α is dispensable for HSC function [100,101]. Germinal centers (GCs) are another example of a physiologically hypoxic environment. During maturation of B cells, the oxygen gradient decreases within the GC [102], possibly as a result of increased oxygen consumption by expanding B cells. Notably, GC hypoxia was found to affect phenotype, proliferation, and class switching of B cells [102,103]. The reproductive organs also show physiological hypoxia. For example, although exact O_2_ tension values in seminiferous tubules remain controversial, the testicular interstitium is hypoxic, showing O_2_ tension values of about 12 to 15 mmHg [104]. Likewise, the vagina is hypoxic under normal conditions [105], and physiological hypoxia in the placenta modulates immune function by protecting the fetus from the maternal immune system; accordingly, HIF dysfunction is associated with placental defects [106]. HIF-1α-mediated gene expression in trophoblasts regulates non-classical class I histocompatibility antigen to prevent attack from natural killer (NK) cells [107], and HIF-1α-mediated programmed cell death 1 ligand 1 (PD-L1) was further found to inhibit T cell responses [108]. The intestinal mucosa is also hypoxic [109], and here, it was shown that physiological hypoxia regulates epithelial barrier function and resident immune cells [110].

### 4.2. Antitumor Immunity

In general, antitumor immunity is similar to persistent antiviral immunity [111]. Although many different immune cells can participate in antitumor responses, T cells are thought to be the most important antitumor immune cells. When tumor antigen is released, antigen-presenting cells (APCs), such as DCs, take-up antigen and migrate into the lymph nodes (LN), where they present their antigen to T cells. Antigen-specific T cells then undergo priming and clonal expansion, and these activated T cells migrate into the tumor area. CD4^+^ T cells orchestrate antitumor immunity and CD8^+^ T cells recognize and directly kill tumor cells [112]. However, tumor cells often poorly express major histocompatibility complex (MHC) class I molecule to escape from CD8^+^ T cell-mediated immunosurveillance [113]. In addition, most tumors have antigens that resemble our self-antigens, thereby inducing tolerance [114]. This limits antitumor T cell activity, and as a result of persistent activation, T cells become exhausted and lose their function. Exhausted CD8^+^ T cells, for example, will express PD-1 on the surface, which binds to PD-L1 on tumor cells or myeloid cells and inhibits T cell function [115,116].

Another class of T cells known as γδ T cells also participate in antitumor immunity and are highly correlated with favorable patient outcomes [117]. These cells can recognize tumor cells via γδTCR or NK-like receptors [118], the upregulation of their ligands of which are induced by transformation and cellular stress [119]. For example, tumor cells highly express NK group 2 member D (NKG2D)-ligands, such as MHC class I polypeptide-related sequence A/B (MICA/B) in humans and retinoic acid early transcript 1 (RAE-1) for mice [120].

Beyond T cells, myeloid cells, including macrophages, monocytes, and neutrophils, can also act as antitumor cells via phagocytosis or the production of inflammatory cytokines [121,122]. However, most tumor-infiltrating myeloid cells are immunosuppressive; these are known as myeloid-derived suppressive cells (MDSCs). MDSCs can suppress antitumor immunity through multiple mechanisms, including interleukin (IL)-10 secretion [123]. Likewise, regulatory T cells (Tregs) also suppress antitumor immune responses [124], and various other immune cells, such as innate lymphoid cells (ILCs) [125], B cells [126], and eosinophils [127], can promote antitumor or protumor responses, depending on the context and environment.

### 4.3. Antitumor Immunity in the GBM

**Figure 3:**
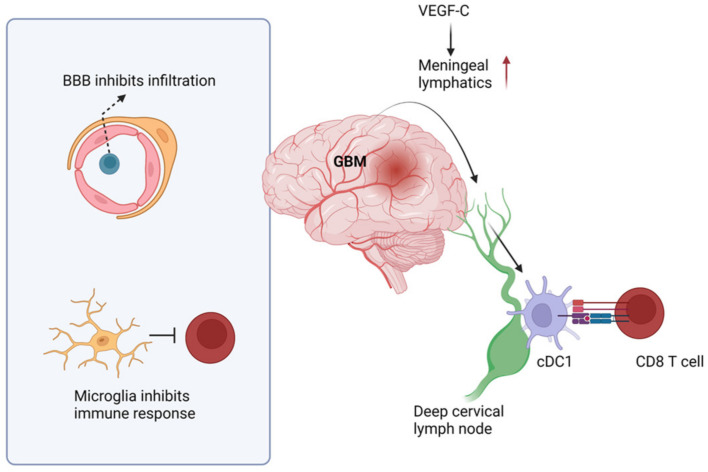
Immune responses in the GBM. GBM antigens are drained by meningeal lymphatics, and classical DC-1s (cDC1s) present antigen to CD8^+^ T cells in the deep cervical lymph node. However, due to the strong blood–brain barrier (BBB), immune cell infiltration into the parenchyma is limited. In addition, the predominant microglia suppress immune responses. However, enhancing lymphatics via VEGF-C treatment has shown promising results to improve survival in animal models.

Unlike peripheral tumors, antitumor immunity against brain tumors has been poorly described. Because of the strong blood–brain barrier (BBB), infiltration of lymphocytes is limited, and thus, the brain is considered to be an immune-privileged organ [128]. Microglia are the predominant immune cells in the brain, although a limited number of other immune cells, such as T cells and mast cells, are also present [129,130]. Likewise, the brain tumor microenvironment is also primarily enriched with microglia and bone marrow-derived macrophages [131]. Due to these unique characteristics, as well as low frequency of neoantigen, antitumor immunity in the brain and responsiveness to immunotherapies is quite poor (Figure 3) [132]. Thus, brain tumors such as GBM are often referred to as “cold tumors” [133].

However, a recent study found that that classical DC-1s (cDC1s) can infiltrate into the GBM area and present antigen to T cells in deep cervical LNs (dcLNs) [134]. This study further suggested that CD141^+^ cDC1s can present antigen in human GBM patients. Regardless, the role of cDC1s and CD8^+^ T cells in GBM is negligible without immunotherapies, such as anti-PD-L1 treatment [135]. Further, our group showed that CD4^+^ and CD8^+^ T cells are dispensable for OS of GBM patients and animals [136]. These studies suggest that although immune responses do occur in the GBM microenvironment, they are too weak to protect host. Consistent with these observations, Song et al. showed that meningeal lymphatics are dampened by GBM progression, suggesting this is one reason for poor anti-GBM immunity [41]. Notably, they further showed that if lymphatics are improved by VEGF-C application, most GBM-bearing animals can survive (Figure 3) [41]. However, extracranial antigen presentation was found to be unable to promote tumor eradication without immunotherapies in a melanoma brain metastasis model [137]. This suggests that antigen presentation in the periphery is unlikely to be sufficient for inducing anti-brain tumor immunity, and further study is needed.

Macrophages and microglia are also important components of anti-GBM immunity. Past in vitro studies have suggested that macrophages can be divided by two groups: M1 and M2. M1 macrophages are related to Th1 responses, whereas M2 macrophages regulate wound healing and Th2 responses [138]. However, recent studies have shown that subsets of macrophages are more complex than previously thought. Tumor-associated macrophages (TAMs), for example, are neither M1- nor M2-like, and rather show mixed phenotypes [139]. Within the tumor microenvironment, most TAMs are M2-like cells; however, proinflammatory TAMs that can phagocytose tumor cells also exist [140]. One well-known mechanism by which this occurs is via the SIRPα–CD47 axis. CD47 is a “do not eat me” signal that inhibits phagocytosis of SIRPα-expressing TAMs. Thus, we can improve phagocytosis by TAMs using CD47 blockade [141]. In addition, the depletion of M2-like TAMs by blocking colony stimulating factor 1 receptor (CSF1R) is also considered to be a promising therapeutic strategy [142]. Conversely, although they are dominant type of immune cells in the brain, the role of microglia in GBM is still unclear. Microglia are usually located at the surrounding edge of the tumor mass rather than inner area [143], but the reason for this is not known, and more studies on microglia in GBM are needed.

NK cells and γδ T cells are also able to kill GBM cells [144,145]. Notably, although in vivo blocking of NK1.1 does not affect OS of GBM-bearing mice [136], another study showed that NK1.1-blockade increases GBM size [146], suggesting an antitumor role for NK cells. Intriguingly, anti-GBM NK cell activity was found to be dependent on the gut microbiota [146]. In addition, NK cells display more potent activity against stem-like GBM cells [144], although direct contact between NK cells and GBM stem cells (GSCs) via αv integrin induces TGF-β-mediated NK cell suppression [147]. Thus, more studies on the role of NK cells in GBM are needed. Other groups have focused on the role of γδ T cells; one murine study showed that Vγ1, Vγ4, and Vγ7 T cells are present in the brain tumor area [148], with Vγ7 comprising the most dominant γ chain. This study further showed that Vδ1, Vδ4, and Vδ6.3 T cells are able to infiltrate into GBM tissue, and in this case, Vδ6.3 is the most prevalent δ chain. In addition, findings from this investigation suggested that γδ T cells are diminished at the terminal stage of tumor progression due to apoptosis, with Vδ6.3^+^ T cells showing the most vulnerability to cell death. Preferential infiltration of Vγ9Vδ2 T cells was also observed in human brain tumor tissue [149], and interestingly, these cells can preferentially kill mesenchymal GBM cells via NKG2D [150]. Further, despite limited investigation, one study suggested that B cells are immunosuppressive within the GBM [151].

### 4.4. The Role of Hypoxia in Anti-GBM Immune Responses

**Figure 4:**
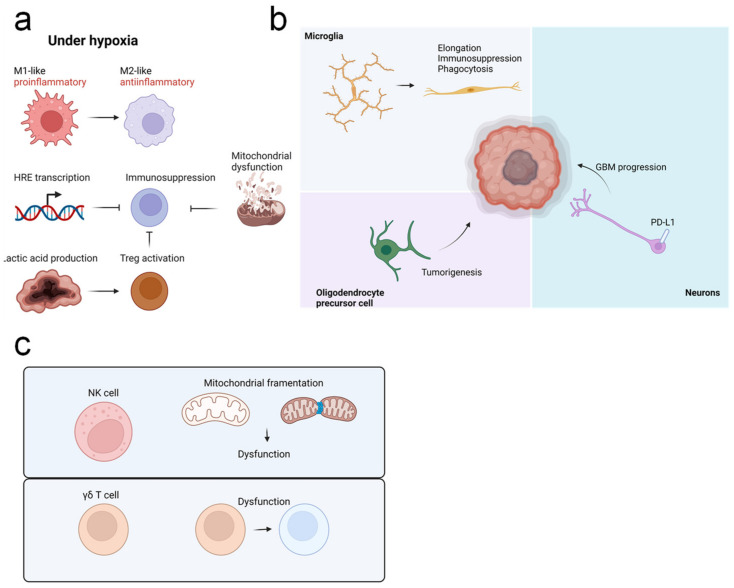
The effect of hypoxia on anti-GBM immune responses. (**a**) Under hypoxia, macrophages preferentially show an immunosuppressive M2-like phenotype, rather than an inflammatory M1-like phenotype. Tumor-infiltrating lymphocytes are suppressed in response to activation of HRE genes and mitochondrial dysfunction, and accumulation of lactic acid supports stability and function of regulatory T cells (Tregs), to further suppress inhibit responses. (**b**) Microglia in the tumor area show an elongated phenotype; these cells are immunosuppressive, with enhanced phagocytosis ability. Oligodendrocyte precursor cells are thought to be a precursor for GBM cells. Connection with neurons also supports GBM progression, whereas PD-L1 expression from neurons is associated with improved prognosis of GBM patients. (**c**) Hypoxia inhibits functions of natural killer (NK) cells and γδ T cells and promotes dysfunction of NK cell mitochondria.

Hypoxia has been shown to affect multiple functions of anti-cancer immune cells. For example, in vitro culture of CD8^+^ T cells under hypoxic conditions promotes reduced levels of proliferation, cytokine production, and cytotoxicity. Melanoma-infiltrating CD8^+^ T cells are also highly exhausted and malfunctional due to severe hypoxia [11]. Similarly, immune cells in GBM core tissue, which primarily comprise M2 macrophages and regulatory T cells, are highly hypoxic (Figure 4a). CD8^+^ T cells within the hypoxic core are also exhausted, and peripheral CD8^+^ T cells cultured in vitro under hypoxic conditions phenocopy CD8^+^ T cells from the GBM core [152].

Expression of HRE genes, including PD-L1, is induced by hypoxia in tumor cells and immune cells [153]. However, in melanoma, hypoxia-induced metabolic stress also inhibits mitochondrial biogenesis in tumor-infiltrating lymphocytes (TILs). Competition between tumor cells and TILs further suppresses the metabolic activity of TILs and inhibits reactivity against immune checkpoint blockade [154], and these immunosuppressive effects are enhanced if hypoxia is chronically persistent [15]. Notably, although PD-1 blockade alone is unable to rescue mitochondrial dysfunction, metabolic reprogramming is sufficient to reverse the exhaustion of TILs [155]. However, TILs in the GBM may be different from those present in subcutaneous tumor models. One study found that modulation of hypoxia using metformin is not sufficient to reinvigorate CD8^+^ T cell responses in a GBM model [136], and further study is needed.

CD4^+^ T cells include various subsets; T helper 1 (Th1) cells resemble CD8^+^ T cells, whereas Tregs show weaker glycolysis and are more oxidative than effector T cells [156]. One study using a B16 melanoma model reported that glucose uptake is closely related to Treg stability, as these cells utilize lactic acid to stabilize their suppressive identity [157]. In the GBM microenvironment, oxidative phosphorylation promotes immunosuppression of Tregs, whereas glycolysis enhances migration (Figure 4a). Further, in a hypoxic microenvironment, Tregs in the GBM use fatty acids for immunosuppression [158], and this is tightly regulated by HIF-1α.

Because hypoxia transiently disrupts the BBB, it may be related to inflammation and inflammation-associated features of the microglia [159]. In Alzheimer’s disease (AD), acute hypoxia induces the M1 transition of microglia [160]. However, hypoxia also inhibits mitochondrial metabolism and promotes the cell cycle arrest of microglia in AD [161]. In GBM tissue, microglia are highly distributed near pseudopalisades and do not escape from hypoxia. In addition, microglia under hypoxia show elongated morphology and increased phagocytosis capability (Figure 4b) [162]. However, the precise role of microglia and hypoxic microglia in GBM is unknown and requires further investigation. Intriguingly, recent studies have shown that other brain-resident cells can participate in GBM progression. For example, oligodendrocyte precursor cells (OPCs) may be associated with GBM progression, and these cells have been proposed as a possible origin of GBM cells [163]. Synaptic and electric communication between GBM cells and neurons also promotes GBM progression [164]. In addition, it was shown that astrocytes suppress immune response in the GBM microenvironment [165]. Because these cell types are also affected by hypoxia, this should be further studied in the context of GBM. Intriguingly, one study reported that neuronal expression of PD-L1, which is known to be regulated by hypoxia, is related to better prognosis of GBM patients [166], thus suggesting a possible role for hypoxic brain cells in GBM progression (Figure 4b).

Hypoxia is also related to dysfunction of NK cells (Figure 4c) [167], as hypoxia-associated mitochondrial fragmentation disrupts NK cell-mediated antitumor immunity [168]. However, the precise effect of hypoxia on NK cell function within the GBM microenvironment remains unclear. Similarly, cytotoxicity of γδ T cells, which are similar to, but distinct from, NK cells, and can kill tumor cells via both γδTCR and NKG2D, is also dampened by hypoxia (Figure 4c) [169]. Further, in the GBM microenvironment, γδ T cells are apoptotic and malfunctional [148], due to the fact that GBM patients receive radiochemotherapy, which also kills γδ T cells. Thus, MGMT-modified γδ T cell therapy might represent an alternative treatment strategy [170]. However, in a murine model, γδ T cells were found to be apoptotic without any treatment [148]. Thus, we expect that the tumor microenvironment suppresses γδ T cell function. Results from our previous study suggest that hypoxia is the critical mechanism mediating suppression of γδ T cells in the GBM [136]. Specifically, we found that when metformin is given to GBM-bearing mice, tumor cell respiration is inhibited, and the remaining oxygen can be utilized by γδ T cells. In addition, adoptive γδ T cell therapy with metformin or HIF-1α is able to prolong overall survival of GBM-bearing mice. Thus, rescuing hypoxia of γδ T cells could be beneficial for γδ T cell-mediated anti-GBM immunity.

## 5. Clinical Perspectives

GBM is one of the most immunologically poor tumor types. Although a number of mechanisms, such as the BBB, participate in immunosuppression, hypoxia is a critical immunosuppressive factor in the GBM microenvironment. Thus, even when immune cells are able to infiltrate into the GBM microenvironment, this hypoxic niche suppresses their antitumor functions. As discussed above, hypoxia inhibits multiple immune cells that are important for antitumor immunity, including CD8^+^ T cells and γδ T cells. Conversely, functions of immunosuppressive cells, such as Tregs and M2 macrophages, are enhanced by hypoxia. As a consequence of this strong immunosuppression, clinical trials assessing the use of immunotherapy for the treatment of GBM have been unsuccessful. An improved understanding of the unique features of anti-GBM immunity is therefore urgently needed to overcome these hurdles and develop effective treatment options. In particular, studies aimed at further investigating the effects of hypoxia on the multiple types of GBM-infiltrating immune cells will help to elucidate the mechanisms by which hypoxia suppresses immune function and determine how this could be overcome. One possible approach is through the use of immunotherapy combined with anti-hypoxic strategies, such as vessel normalization. Alternatively, cell therapy using engineered hypoxia-resistant immune cells may be another option for next-generation immunotherapy against hypoxic tumors.

## 6. Conclusions

Hypoxia is a classic hallmark of tumors [5], and GBM is one of the most hypoxic tumors. Hypoxia affects multiple aspects of GBM biology and pathology, including vasculature, invasiveness, resistance to drugs, and antitumor immune responses [42,171]. Critically, hypoxia-driven invasion and angiogenesis are highly associated with poor prognosis, and hypoxia-mediated resistance to conventional therapies, including chemotherapy and radiation, is a significant hurdle when caring for GBM patients. Furthermore, recently developed immunotherapies are also not effective against GBM, as it is considered to be a “cold tumor”, with low neoantigen levels and poor immune cell infiltration [132]. There are many reasons why GBM is a “cold tumor”, and hypoxia is one critical factor that promotes immunosuppression within the GBM microenvironment [61]. Although the detrimental role of hypoxia in immune cell function has been well-studied, the precise impact of hypoxia on anti-GBM immunity is unclear. In particular, CD8^+^ T cells are known to be suppressed by hypoxia, but unlike in other tumors, a re-oxygenation strategy was not effective for restoring the CD8^+^ T cell function in GBM. In contrast, improving the oxygen metabolism of γδ T cells was sufficient to increase the survival period of GBM-bearing animals, suggesting a critical role for these cells [136]. Thus, the effect of hypoxia on various immune cell types within the GBM is a critical area of investigation, as targeting hypoxia may be beneficial for improving the efficacy of conventional therapy and immune responses against this deadly tumor.
